# Immortalized N/TERT keratinocytes as an alternative cell source in 3D human epidermal models

**DOI:** 10.1038/s41598-017-12041-y

**Published:** 2017-09-19

**Authors:** Jos P. H. Smits, Hanna Niehues, Gijs Rikken, Ivonne M. J. J. van Vlijmen-Willems, Guillaume W. H. J. F. van de Zande, Patrick L. J. M. Zeeuwen, Joost Schalkwijk, Ellen H. van den Bogaard

**Affiliations:** 1 Department of Dermatology, Radboud Institute for Molecular Life Sciences (RIMLS), Radboud University Medical Center (Radboudumc), PO BOX 9101, 6500 HB Nijmegen, The Netherlands; 2 Department of Human Genetics, Radboud University Medical Center (Radboudumc), PO BOX 9101, 6500 HB Nijmegen, The Netherlands

## Abstract

The strong societal urge to reduce the use of experimental animals, and the biological differences between rodent and human skin, have led to the development of alternative models for healthy and diseased human skin. However, the limited availability of primary keratinocytes to generate such models hampers large-scale implementation of skin models in biomedical, toxicological, and pharmaceutical research. Immortalized cell lines may overcome these issues, however, few immortalized human keratinocyte cell lines are available and most do not form a fully stratified epithelium. In this study we compared two immortalized keratinocyte cell lines (N/TERT1, N/TERT2G) to human primary keratinocytes based on epidermal differentiation, response to inflammatory mediators, and the development of normal and inflammatory human epidermal equivalents (HEEs). *Stratum corneum* permeability, epidermal morphology, and expression of epidermal differentiation and host defence genes and proteins in N/TERT-HEE cultures was similar to that of primary human keratinocytes. We successfully generated N/TERT-HEEs with psoriasis or atopic dermatitis features and validated these models for drug-screening purposes. We conclude that the N/TERT keratinocyte cell lines are useful substitutes for primary human keratinocytes thereby providing a biologically relevant, unlimited cell source for *in vitro* studies on epidermal biology, inflammatory skin disease pathogenesis and therapeutics.

## Introduction

*In vitro* cultured 3D human skin equivalents (HSEs) and human epidermal equivalents (HEEs) (also known as organotypic skin models) are nowadays often used for studies in the fields of cell biology, tissue engineering, dermatology, and toxicology. These 3D skin models are preferred over the conventional monolayer cultures as they faithfully mimic the *in vivo* epidermis and skin barrier function and thereby serve as alternatives for experimental animal testing^1^. To meet the growing demand for these *in vitro* 3D human organotypic skin cultures, large quantities of primary keratinocytes are required. Human primary keratinocytes are preferably isolated from adult skin excised during plastic surgery (e.g. abdominal wall or breast reduction). Unfortunately, the availability of donor skin is limited and the short *in vitro* lifespan and inter-donor variation are disadvantages of the use of human primary keratinocytes. Immortalized keratinocyte cell lines could therefore provide an unlimited cell source and solution to the aforementioned issues.

The HaCaT cell line, a spontaneously immortalized human keratinocyte cell line, is widely used in keratinocyte monolayer culture models and also described for the development of organotypic skin models^2^. HaCaT cells respond to differentiation-promoting stimuli, such as contact inhibition and high calcium concentrations in the culture medium^2–4^. However, the transcriptional expression pattern of cornified envelope-associated proteins, such as filaggrin, loricrin and involucrin is abnormal compared to normal human primary keratinocytes^5,6^ and karyotyping of HaCaT cells shows aneuploidy^5^. Wound regeneration and eczematous organotypic models with HaCaT cells have been reported^6–10^, however, epidermal stratification is abnormal, aberrant epidermal differentiation protein expression is observed and a *stratum corneum* is lacking in these HaCaT-derived 3D skin models.

In 2000, the Rheinwald laboratory developed two immortalized keratinocyte cell lines, called the N/TERT cell lines (N/TERT1 and N/TERT2G), generated by transduction of human primary keratinocytes with the human telomerase reverse transcriptase (hTERT) gene and by the spontaneous loss of the pRB/p16^INK4a^ cell cycle control mechanism^11^. This cell line was shown to have normal differentiation characteristics in monolayer and organotypic skin models. Remarkably, the use of the HaCaT cell line prevailed in experimental dermatological research and to date a limited number of studies have used N/TERT keratinocytes. (See Table 1 for studies using organotypic skin models and Supplemental Table 1 for studies using N/TERT cells in conventional monolayer cultures). Unfortunately, a direct comparison of N/TERT keratinocytes to primary human keratinocytes is lacking and this may have hampered the wide implementation of the N/TERT keratinocyte cell line in biomedical research. In addition, in toxicological research, the HEE models: SkinEthic^26^, EpiDerm^27^, and EPISKIN^28^ are accepted alternatives for skin irritation and corrosion testing. Herein, the N/TERT cell line could be a valuable substitute for primary keratinocytes, however studies on N/TERT cells in HEE models are scarce^17,18,21–23^ and a thorough characterization with regard to epidermal differentiation and response to inflammatory mediators is lacking.

**Table 1:** Studies using *in vitro* 3D reconstructed skin generated from N/TERT keratinocytes.

3D skin model	Dermal matrix	F	Epidermal morphology	Field of study	Reference
HSE	Collagen type I	Y	Stratified epidermis, sc present	Cell line development	Dickson *et al*.^11^^$^
HSE	Collagen type I	Y	Stratified epidermis, sc present	Cell line development	Rheinwald *et al*.^12^
HSE	DED	Y	Stratified epidermis, sc present	Epidermal biology	Wan *et al*.^6^
HSE	DED	Y	Stratified epidermis, sc present	Skin barrier	Man *et al*.^13^
HSE	Collagen type I	Y	Stratified epidermis, sc present	Epidermal biology	Dabelsteen *et al*.^14^
HEE	None	N	Stratified epidermis, sc present	UV radiation, DNA damage	Bertrand-Vallery *et al*.^15^
HSE	Collagen type I	N	Stratified epidermis, sc present	Human papillomavirus	Lazic *et al*.^16^
HEE	None	N	Stratified epidermis, sc present	Epidermal biology	Robertson *et al*.^17^
HSE	Collagen type I	Y	Stratified epidermis, sc present	Skin barrier	Van Drongelen *et al*.^18^
HSE	Collagen type I	Y	Multilayer of cells, sc lacking	UV radiation, DNA damage	Harrison *et al*.^19^
HSE	Collagen type I	Y	Multilayer of cells, sc present	Ionizing radiation, DNA damage	Acheva *et al*.^20^
HSE	Collagen type I	Y	Stratified epidermis, sc present	Skin barrier	Van Drongelen *et al*.^21^
HEE	None	N	Stratified epidermis, sc present	Epidermal biology	Van Drongelen *et al*.^22^
HEE	None	N	Stratified epidermis, sc present	Skin sensitization	Alloul-Ramdhani *et al*.^23^
HSE	Matriderm	Y	Stratified epidermis, sc present	Epidermal biology	Reijnders *et al*.^24^
HSE	Collagen type I	Y	Stratified epidermis, sc present	Ionizing radiation, inflammation	Acheva *et al*.^25^

^$^Initial paper describing the N/TERT cell line development; HSE: human skin equivalent; HEE: human epidermal equivalent; DED: de-epidermized dermis; F: Fibroblasts present Yes/No; UV: ultraviolet; sc: *stratum corneum*.

Earlier studies by our group and others showed that the addition of T-helper-(Th)1 or Th2 cytokines to 3D skin models induce a phenotype in keratinocytes that is similar to psoriasis (PS) or atopic dermatitis (AD), respectively^29–31^. PS and AD are highly prevalent multifactorial chronic inflammatory skin diseases of which PS is considered to be Th1-Th17 immune cell mediated, while in AD the Th2 cells and cytokines prevail. Current treatment is often aimed at general immunosuppression, which is associated with side effects. Therefore, an unmet medical need exists for novel targeted, therapeutic approaches. *In vitro* inflammatory skin disease models are considered valuable tools to study disease pathogenesis and can be used in a pre-clinical drug screening setting. However, the large-scale production and implementation of these models is lacking and this may be due to the limited source of primary keratinocytes. The N/TERT keratinocyte cell lines could provide a solution herein, but only if their response to inflammatory mediators such as Th1, Th2, and Th17 cytokines is similar to that of primary keratinocytes.

To illustrate the potential of N/TERT keratinocytes for studies on epidermal biology, inflammatory skin disease pathogenesis and therapeutics, we compared the mRNA and protein expression of two N/TERT keratinocyte cell lines to that of human primary epidermal keratinocytes in monolayer cultures. Next, we generated a N/TERT-HEE model and characterized mRNA and protein expression, and physical barrier properties of this 3D epidermal model by a comparison to HEEs generated from primary keratinocytes. PS-associated (Th1 and Th17) or AD-associated (Th2) cytokines were used to generate 3D human skin inflammation models and we validated disease markers in both models by known anti-inflammatory drugs.

## Results

### Karyotyping and short tandem repeat analysis of N/TERT keratinocytes

The N/TERT1 and N/TERT2G keratinocytes were karyotyped to assess their ploidy according to standard procedures. N/TERT2G is diploid (46, XY) and N/TERT1 is diploid with an additional chromosome 20 (47, XY, +20) (Supplemental Fig. 1). To assess potential contamination with other cells, short tandem repeat (STR) analysis was performed by quantitative fluorescence (QF)-PCR using 21 specific targets on chromosome 13, 18, 21, and both sex chromosomes X and Y (Supplemental Fig. 2). All targets, except D18S391, are informative and rule out cellular contamination. Only N/TERT1 is shown, as both N/TERT keratinocytes showed identical results.

### Differentiation kinetics and dynamics of N/TERT keratinocytes in conventional cultures

**Figure 1:**
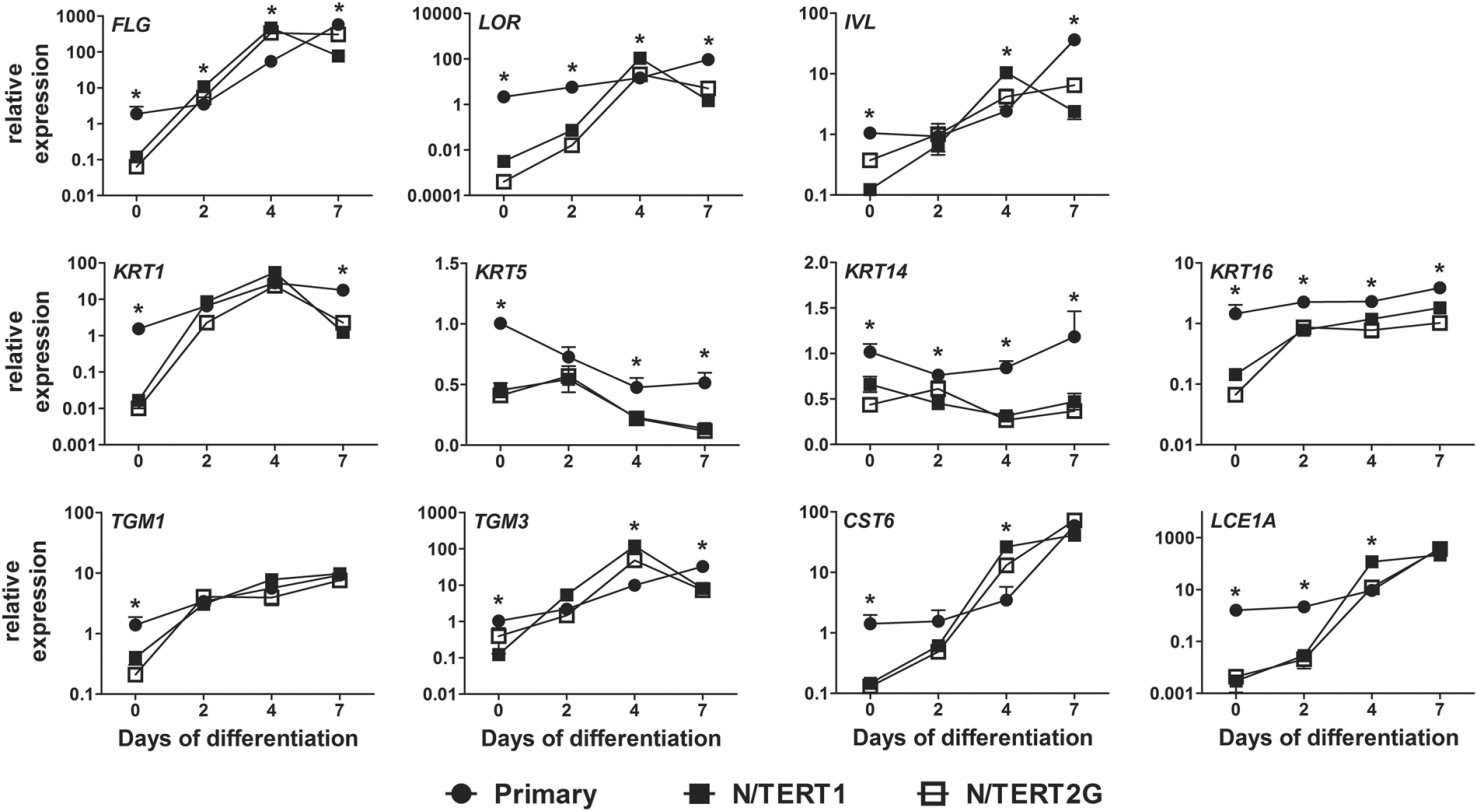
N/TERT keratinocytes express terminal differentiation genes. mRNA expression of epidermal differentiation genes by N/TERT keratinocyte monolayer cultures (N = 3 for N/TERT 1 and N/TERT2G) were compared to primary keratinocytes (N = 6 donors). Bars represent mean ± SEM. *p < 0.05 relative to primary keratinocyte at the same time point.

To evaluate the differentiation dynamics of the N/TERT1 and N/TERT2G keratinocytes we performed a time course and compared N/TERT keratinocytes to primary human keratinocytes. We analysed epidermal differentiation gene expression levels in monolayer cultures up to seven days of differentiation. Relative expression levels of N/TERT keratinocytes were normalized to primary keratinocytes, giving an insight into the basal expression levels and expression patterns of N/TERT keratinocytes at different time points (Fig. 1, t = 0, 2, 4 and 7 days of differentiation). At confluency, which is the start of the differentiation phase (t = 0) due to cell-cell contact inhibition, the basal expression level of all investigated epidermal genes was significantly lower in N/TERT keratinocytes (terminal differentiation genes: *filaggrin* (*FLG)*, *loricrin* (*LOR)*, *involucrin* (*IVL), late cornified envelope (LCE) 1A*; *keratins* (*KRT*): *KRT1*, *KRT5*, *KRT14*, *KRT16*; and genes involved in cornification and desquamation: *transglutaminase* (*TGM) 1*, *TGM3*, *cystatin M/E* (*CST6*)). Expression levels increased at day 2 and at day 4 the expression of *FLG*, *LOR*, *IVL*, *TGM3*, *CST6*, *LCE1A* was significantly higher in the N/TERT keratinocytes as compared to primary keratinocytes, indicating a significantly stronger induction rate of N/TERT keratinocytes from day 0 to day 4. The N/TERT keratinocyte gene expression levels reached a maximum at day 4 and dropped below the primary keratinocyte levels at day 7. Primary keratinocytes tend to reach their highest expression levels not earlier than at day 7. These results indicate that the N/TERT keratinocytes respond very strongly to differentiation stimuli and reach similar expression levels of terminal differentiation genes as primary keratinocytes, albeit at an earlier time point in culture.

### Human epidermal equivalents can be generated from human N/TERT keratinocytes

**Figure 2:**
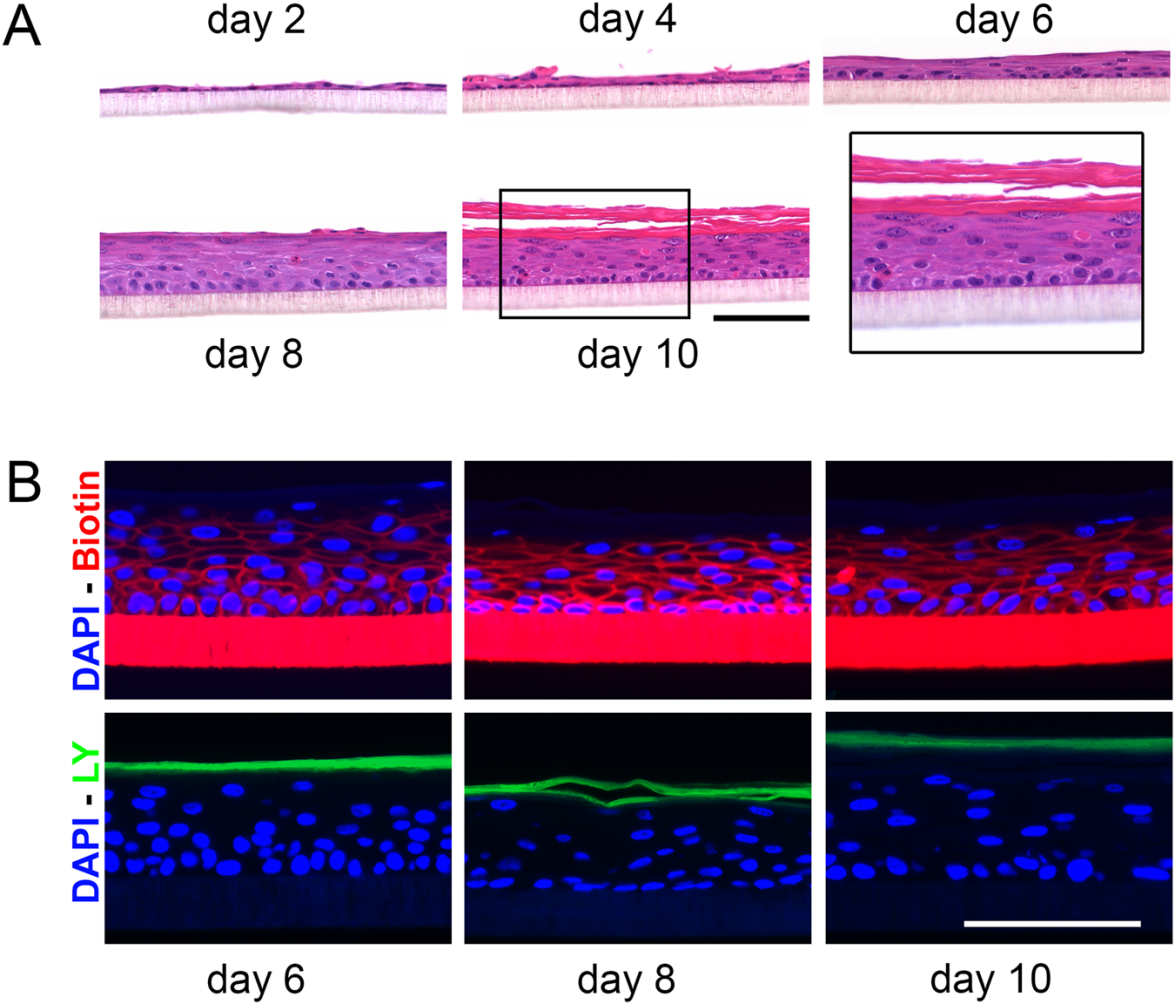
N/TERT1 keratinocytes are suitable for culture in HEE models. (**A**) Haematoxylin Eosin (HE) staining of N/TERT1 keratinocytes in the HEE model system while developing from day 2 to day 10 of air exposure. (**B**) Epidermal barrier properties of the HEE constructs by biotin penetration (red) and Lucifer yellow penetration (green). Scale bar = 100 µm.

To study the potential of N/TERT keratinocytes for the implementation in 3D skin model development, we optimised N/TERT HEE culture protocols (Supplemental Fig. 3) and studied the epidermal stratification and functional characteristics herein. Briefly, using K-SFM medium (as compared to DMEM which is used in primary keratinocyte HEE cultures) during the proliferation phase of the culture period and CnT-PR-3D medium supplemented with DMEM (60:40) in the differentiation phase resulted in most optimal epidermal morphology (Supplemental Fig. 4A). Furthermore, the seeding of approximately 1·10^5^ N/TERT cells (Supplemental Fig. 4B) directly from liquid nitrogen (passage 0) or cultured cells until passage 4 (Supplemental Fig. 4C) generated high quality HEEs where N/TERT keratinocytes generated a multilayered epidermis with presence of a *stratum granulosum* and a basket weave *stratum corneum* in approximately 10 days of air-exposed culture (Fig. 2A). Using immunohistochemistry we studied the expression of epidermal differentiation protein FLG, the expression of the proliferation marker Ki67, and the expression of anti-microbial peptides human beta defensin 2 (hBD2), and skin-derived antileukoprotease (SKALP). We compared the expression patterns with HEE cultures generated from primary human keratinocytes and found highly similar protein expression patterns, apart from a lower expression of FLG and stronger expression of SKALP in the N/TERT-HEEs (Supplemental Fig. 4D,E). To analyse the *stratum corneum* barrier characteristics of N/TERT keratinocyte-based 3D HEEs we performed ‘outside-in’ (Lucifer Yellow (LY)) and ‘inside-out’ (EZ-Link Sulfo-NHS-LC-LC-Biotin) tracer penetration assays, as previously reported^32^. We found restricted dye penetration from day 6 of the air-liquid interphase culture onwards (Fig. 2B). These findings correspond to data obtained with HEEs generated from primary human keratinocytes^32^.

### Human N/TERT and primary keratinocytes respond similar to pro-inflammatory cytokine stimulation in conventional monolayer cultures

**Figure 3:**
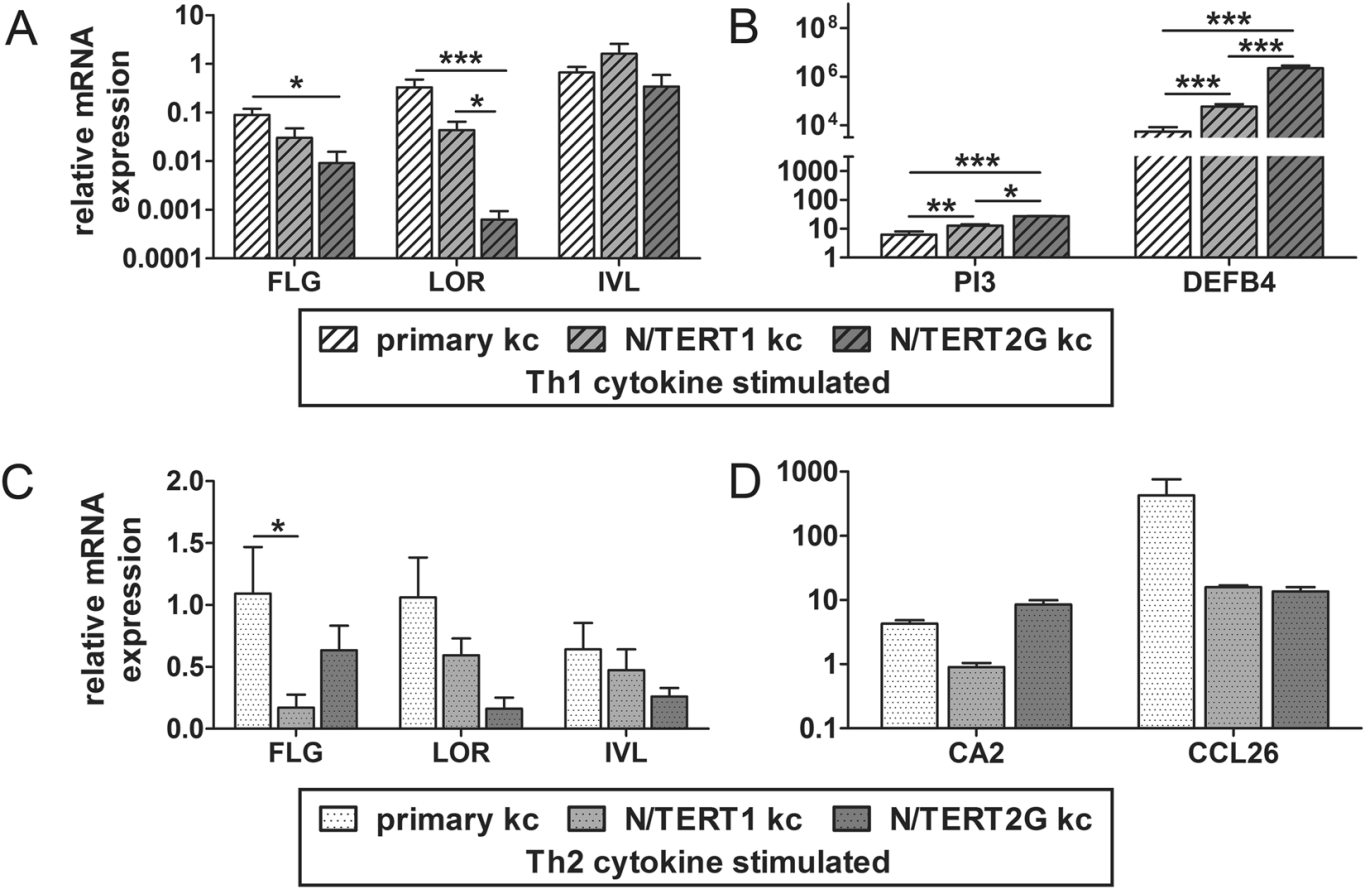
N/TERT keratinocytes and primary keratinocytes respond similar to pro-inflammatory cytokine stimulation in conventional monolayer cultures. (**A**) Terminal differentiation genes and (**B**) psoriasis marker mRNA expression of monolayer N/TERT keratinocytes and primary keratinocytes after stimulation with Th1 cytokines (IL-1α, TNFα, and IFNγ). (**C**) Terminal differentiation genes and (**D**) AD marker mRNA expression of monolayer N/TERT keratinocytes and primary keratinocytes after stimulation with Th2 cytokines (IL-4 and IL-13). Bars represent mean ± SEM. *p < 0.05 **p < 0.01 ***p < 0.001 relative to primary keratinocytes.

Next, the expression levels of terminal differentiation genes and inflammatory disease marker genes were analysed in monolayer N/TERT keratinocytes that were stimulated with pro-inflammatory Th1 or Th2 cytokines. Upon stimulation with Th1 cytokines (TNFα, IFNγ, and IL-1α) the expression levels of *FLG* and *LOR* decreased (normalized to unstimulated keratinocytes) and in particular in N/TERT2G keratinocytes this downregulation was significantly stronger as compared to primary keratinocytes (Fig. 3A). The expression of psoriasis-related host defence genes *PI3* and *DEFB4* ^33–35^ (encoding SKALP/elafin and hBD2, respectively) was increased in both primary and N/TERT keratinocytes, but N/TERT keratinocytes showed a significantly stronger upregulation and this was most pronounced in N/TERT2G cultures (Fig. 3B). After stimulation with Th2-cytokines (IL-4 and IL-13), we observed a minor decrease (only statistically significant for FLG) in *FLG*, *LOR* and *IVL* expression in N/TERT keratinocytes (Fig. 3C), while markers for AD, *C-C motif chemokine ligand 26* (*CCL26*) and *carbonic anhydrase 2* (*CA2*)^30,36^, were found to be upregulated (Fig. 3D). Overall, the N/TERT keratinocytes appear more responsive to Th1 cytokine stimulation than primary keratinocytes. Upon addition of Th2 cytokines, N/TERT1 keratinocytes express less *CA2* than primary keratinocytes and N/TERT2G keratinocytes. *CCL26* is upregulated by both N/TERT cell lines and the primary keratinocytes upon Th2 cytokine addition, albeit a stronger induction rate of primary keratinocytes. These differences are, however, not statistically significant due to the high interdonor variability of the primary keratinocytes.

### Psoriasis-like HEE model (PS-HEE) generated from N/TERT keratinocytes

We explored the potential of N/TERT keratinocytes for generating *in vitro* inflammatory skin models for psoriasis by the addition of pro-inflammatory cytokines to N/TERT-HEEs. We added psoriasis-associated cytokines of Th1 (TNFα, IL-6, and IL-1α) and Th17 (IL-17 and IL-22) origin to N/TERT1 HEEs during the last three days of the air-liquid interphase culture. We furthermore aimed to rescue the disease phenotype by the addition of all trans retinoic acid (ATRA), an anti-psoriatic drug, to the culture medium supplemented with Th1 or Th17 cytokines as previously described for primary keratinocyte 3D skin models^31,37^.

**Figure 4:**
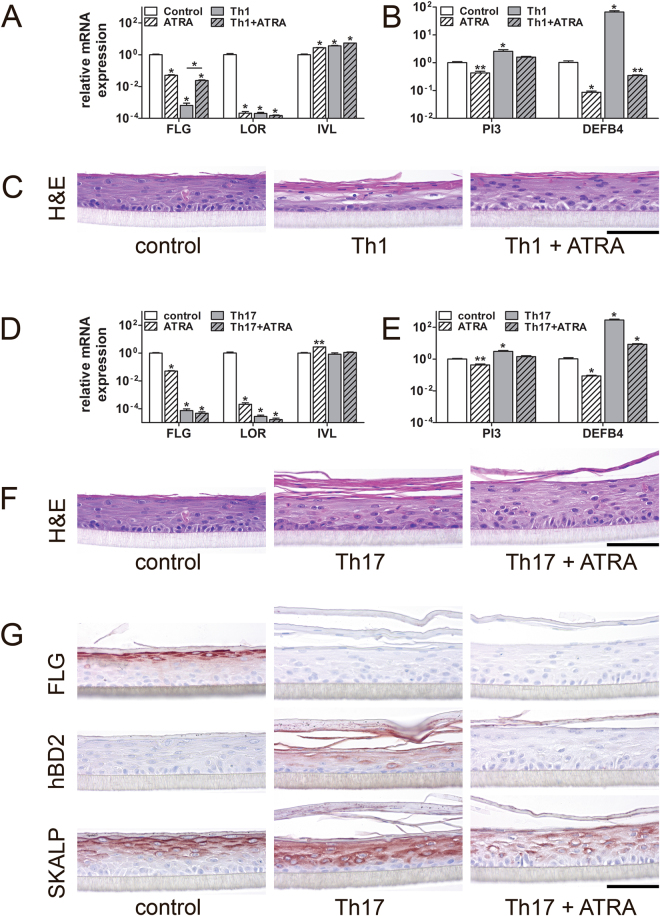
N/TERT1 keratinocytes are suitable to generate a psoriasis-like HEE (PS-HEE) disease model. (**A**,**B**) N/TERT HEEs were harvested for mRNA expression analysis and (**C**) morphological analysis after stimulation with Th1 cytokines (TNFα, IL-6, and IL-1α) and rescued by addition of all trans retinoic acid (ATRA). (**D**,**E**) N/TERT HEEs were harvested for mRNA expression analysis and (**F**,**G**) morphological analysis after stimulation with Th17 cytokines (IL-17 and IL-22) and rescued by addition of all trans retinoic acid (ATRA). Images are representative of N = 3 N/TERT HEE experiments. Bars represent mean ± SEM. *p < 0.001 **p < 0.01 relative to control unstimulated keratinocytes. Scale bar = 100 µm.

Upon Th1 and Th17 cytokine stimulation we found a strong and significant decrease in the expression of *FLG* and *LOR*, while *IVL* expression was upregulated by Th1 cytokines (Fig. 4A,B). ATRA treatment successfully rescued *FLG* expression levels after the Th1 cytokine stimulation, but also significantly altered *FLG*, *LOR* and *IVL* expression alone. Both Th1 and Th17 cytokines significantly induced the expression of the host defence genes *PI3* and *DEFB4* (Fig. 4B,E). This closely resembles the transcriptional expression patterns that are found in psoriasis lesions *in vivo* ^38–40^. ATRA treatment reduced the expression of *PI3* and *DEFB4* in Th1 and Th17 cytokine stimulated N/TERT-HEEs to levels that are close to the control situation (Fig. 4B,E).

Th1 and Th17 cytokines affected the epidermal morphology as haematoxylin eosin stainings showed parakeratosis (retained nuclei in the *stratum corneum*), thickening of the *stratum corneum* and the absence of a *stratum granulosum* in these cultures (Fig. 4C,F), all features of psoriatic skin. Treatment with ATRA improved the epidermal morphology in both cytokine models (Fig. 4C,F). The changes in epidermal morphology were accompanied by lowered protein expression levels of the terminal differentiation protein FLG, while psoriasis markers hBD2 and SKALP were upregulated. ATRA treatment lowered hBD2 and SKALP expression but did not restore FLG protein levels (Fig. 4G and Supplemental Fig. 5). These results indicate that N/TERT cells can be used to generated PS-HEEs and we were able to validate the model by the treatment with a known anti-psoriatic drug.

### Atopic dermatitis-like HEE model (AD-HEE) generated from N/TERT keratinocytes

**Figure 5:**
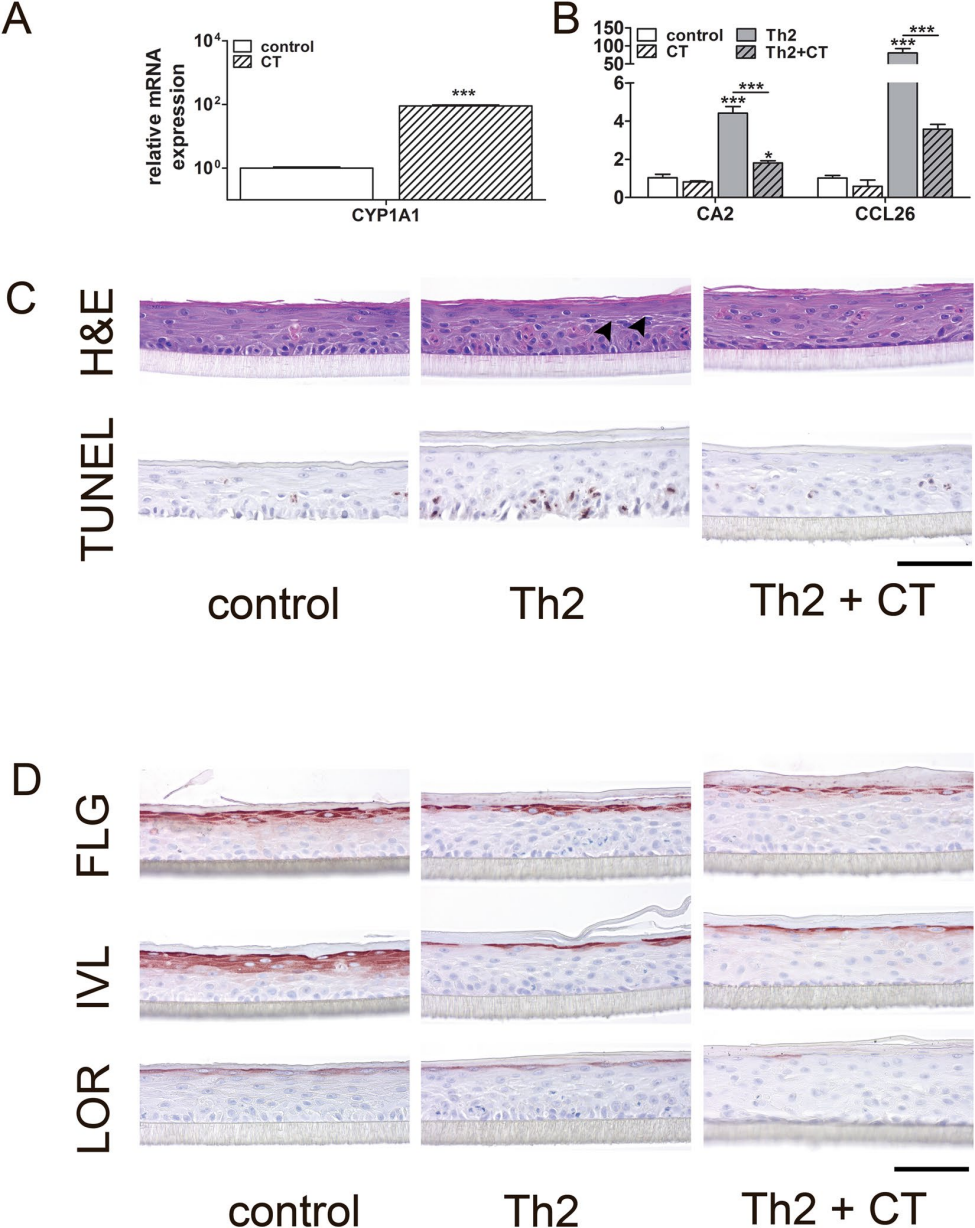
N/TERT1 keratinocytes can be used to generate a atopic dermatitis-like HEE (AD-HEE) disease model. (**A**) mRNA expression of N/TERT1 HEEs after coal tar stimulation. Activation of the aryl hydrocarbon receptor (AHR) is measured via the induction of the AHR target gene, *CYP1A1*. (**B**) N/TERT HEEs were harvested for mRNA and (**C**) morphological analysis after stimulation with Th2 cytokines (IL-4 and IL-13) and rescued by addition of coal tar extract. (**D**) Protein expression was visualised by immunohistochemistry. Bars represent mean ± SEM. *p < 0.05 **p < 0.01 ***p < 0.001. Scale bar = 100 µm.

We used the Th2 cytokines IL-4 and IL-13 to induce histopathological and molecular hallmarks of AD. These include acanthosis, spongiosis, and apoptosis^41–43^ and disease-associated gene and protein expression patterns in HEEs generated from N/TERT1 and N/TERT2G keratinocytes. To explore the potential of the N/TERT atopic dermatitis-like inflammatory HEE model (AD-HEE) model for pre-clinical drug screening, we used coal tar as a therapeutic model agent. Coal tar therapy is an ancient treatment for AD and psoriasis^44,45^ and we recently uncovered its molecular mechanism of action, namely activation of the aryl hydrocarbon receptor (AHR)^46^. The well known AHR target gene, cytochrome p450 family member 1A1 (*CYP1A1*)^47^ is strongly induced by primary keratinocytes after coal tar exposure^48^ and N/TERT keratinocytes respond in a similar manner (Fig. 5A, Supplemental Fig. 6A). Other AHR target genes like 2,3,7,8-tetrachlorodibenzo-*p*-dioxin (TCDD) inducible poly(ADP-ribose) polymerase (*TIPARP*), cytochrome p450 family member 1B1 (*CYP1B1*), aryl hydrocarbon receptor repressor (*AHRR*) (Supplemental Fig. 6A) and epidermal differentiation genes, *IVL* and *HRNR* (Supplemental Fig. 6B) were also induced in N/TERT keratinocytes after coal tar stimulation while the expression of AD-related genes^30,49^ *CCL26* and *CA2* were significantly upregulated in the N/TERT AD-HEE (Fig. 5B). Epidermal morphology of N/TERT AD-HEEs showed signs of spongiosis in the suprabasal layers (intercellular oedema, seen as white spaces between adjacent cells in the HE staining) (Fig. 5C, panel HE, arrowheads) and apoptotic basal cells (Fig. 5C, panel TUNEL). Coal tar treatment reduced the histopathological AD hallmarks but did not rescue the Th2-cytokine mediated downregulation of epidermal differentiation proteins, FLG, IVL and LOR (Fig. 5D).

## Discussion

Immortalized human cell lines have been instrumental for scientists to validate data obtained in animal models and for the translation to human biology and disease. Yet, the demand for complex tissue-like cell culture models to better represent the *in vivo* situation is ever rising. In skin research, organotypic 3D skin models are nowadays considered the gold standard, however, the use of primary human keratinocytes is herein required to obtain optimal tissue morphology. High costs for commercially available primary cells and the large quantity of cells needed to generate 3D skin models have directed research groups to isolate primary keratinocytes from surplus skin. Unfortunately, the availability of donor skin is limited and high inter-donor variability requires a multitude of biological replicates for a good reproducibility of results. Immortalized N/TERT keratinocytes are diploid (Supplemental Figs 1 and 2) with the exception of an additional chromosome 20 in the N/TERT1 keratinocyte cell line. A gain of chromosome 20 is related to increased proliferative potential and often observed in immortalized cells and in several cancer cell lines^48,50–54^. Nevertheless, both N/TERT keratinocytes mimic major characteristics of primary human keratinocytes and have superior qualities over the most widely used HaCaT (aneuploid^5^) keratinocyte cell line with regard to epidermal differentiation and the formation of a stratified epithelium. The herein provided literature overview of research utilizing N/TERT keratinocytes (Table 1 and Supplemental Table 1) enables others to determine the suitability of the N/TERT keratinocytes for their research.

Besides the use of HaCaT cells, the use of the Rho kinase inhibitor, Y-27632, was demonstrated to generate immortalized epithelial cell lines^55^. Although it is possible to generate immortalized primary foreskin keratinocytes, and to prolong the life span of adult keratinocytes through the use of Y-27632^55,56^, cells require the inhibitor throughout the cell culture. Since Rho kinases are vital for many cellular processes, potential off-target effects may occur. In addition, the adult keratinocytes are not stably immortalized by the Rho kinase inhibition and senescence occurs after five to eight passages. Therefore, this method seems less suitable for experiments that require extensive cell handling, sorting and prolonged subcultures.

The initial study first describing the N/TERT1 and N/TERT2G cell lines^11^ already showed the potential of N/TERT keratinocytes to retain normal growth and differentiation characteristics *in vitro*. These data together with the herein presented direct comparison to human primary keratinocytes clearly indicate that N/TERT keratinocytes largely behave like primary keratinocytes. N/TERT keratinocytes tend to differentiate quicker than primary keratinocytes as shown by the gene expression peaks at day 4 of differentiation in N/TERT keratinocytes and at day 7 of differentiation in primary human keratinocytes (Fig. 1). Therefore, when using N/TERT keratinocytes in a monolayer culture system, one should be cautious about the time of harvesting when differentiation status is critical for the experimental outcome.

Since the introduction of the N/TERT cell line seventeen years ago, we only found sixteen papers describing the use of these cells in the development of organotypic human skin models (Table 1). The majority have used a dermal substrate based on collagen, fibroblasts or human decellularized dermis. Only four studies^15,17,22,23^ have generated an epidermis only model from N/TERT keratinocytes, similar to the commercially available SkinEthic^26^, EpiDerm^27^, and EPISKIN^28^ whom are generated from primary human keratinocytes. A thorough characterization of epidermal morphology, differentiation, and barrier function of N/TERT HEEs was still lacking, hence the current study. We found that both N/TERT cell lines (N/TERT1 and N/TERT2G) generate HEEs that are similar to primary keratinocyte-based HEEs. The only protein that is absent in normal skin, yet detected in N/TERT HEEs is SKALP. This antimicrobial protein is involved in keratinocyte host defence responses and highly upregulated in psoriasis skin^57^. The expression of SKALP in organotypic skin cultures is however not unusual^31^. N/TERT keratinocytes are derived from primary neonatal foreskin keratinocytes, which are known for higher expression levels of SKALP in comparison to primary adult keratinocytes^31^.

We found the basket weave *stratum corneum* of N/TERT HEEs to form a functional barrier to low molecular weight tracers (Fig. 2A,B) that are widely used for studying the outside-inside and inside-outside barrier function of the *in vitro* reconstructed epidermis^32,58^. The *stratum corneum* lipid composition and ultrastructure of N/TERT HSEs has been studied by the group of El Ghalbzouri^21^, showing a high similarity to HSEs generated from primary human keratinocytes. Although we cannot exclude potential differences between N/TERT and primary keratinocytes for the penetration of other tracer molecules or microbial components, and differences between the *stratum corneum* formation in HSE *versus* HEE models may occur, N/TERT keratinocytes show a great potential substituting primary keratinocytes in 3D reconstructed models for normal skin. Important to note is that the HEE culture model system should not be considered as a full thickness skin model, but as an epidermal equivalent. The inert plastic filter obviously does not replicate the functions of the native basement membrane and the absence of a dermal matrix also precludes investigations on dermal-epidermal interactions. Other groups already showed the feasibility of generating full thickness skin equivalents using N/TERT cells^18,21,23^, therefore we focussed on the application of the N/TERT cells in the epidermal only models and models for inflammatory skin diseases. Altogether the N/TERT keratinocytes are now described in different culture models for human skin, using diverse culture protocols by various research groups. This clearly indicates the good reproducibility of data obtained from N/TERT cells and strengthens the notion that this cell line is a powerful tool in experimental dermatology research.

To date, no studies investigated the feasibility of using the N/TERT-HEEs for studies on inflammatory skin diseases. Psoriasis and atopic dermatitis are highly prevalent, inflammatory skin disease of multifactorial aetiology but with a distinct disease pathophysiology. Increased proliferation, lack of a *stratum granulosum* and parakeratosis (retention of nuclei in the *stratum corneum* resulting from incomplete keratinocyte maturation^59^) are morphological hallmarks of psoriatic skin^60–62^. Epithelium-specific genes like *DEFB4*, *PI3*, *S100A7*, and *KRT16* and their corresponding proteins are highly expressed in psoriatic epidermis and are considered disease markers^57,63^. The immunological component of psoriasis is marked by high levels of pro-inflammatory cytokines and chemokines, including interferon-γ, interleukins 1, 6, 17, and 22 and tumor necrosis factor (TNF)α^64–69^. Addition of the abovementioned pro-inflammatory cytokines to human primary keratinocytes is frequently described to generate *in vitro* models for psoriatic skin^31,70–74^. We found that the addition of Th17 cytokines, IL-17 and IL-22, to the N/TERT-HEEs most closely resembled psoriasis skin: presence of parakeratosis, lack of a *stratum granulosum* (and thus low levels of FLG and LOR), and induced levels of hBD2 and SKALP (Fig. 4G). The addition of Th1 cytokines (TNFα, IL-6, and IL-1α) resulted in similar effects, yet these cytokines had more deleterious effects on the epidermis resulting in less optimal epidermal morphology (Supplemental Fig. 5). As we intended to compare the responses of N/TERT keratinocytes to primary keratinocytes, we applied the cytokine cocktails used for primary keratinocytes and did not optimise concentrations or combinations which may improve the epidermal morphology of the Th1 model. Other psoriasis hallmarks, like the presence of rete ridges, microabcesses of Munro, pustules of Kogoj, and aberrations of dermal capillaries^60,75^ can obviously not be studied in our model, as it lacks a dermal compartment with immune cells and blood vessels. Of note, the N/TERT PS-HEE does not show acanthosis or hyperproliferation. Since the majority of primary keratinocyte-derived organotypic skin models lack these features, this seems a problem inherent to the skin- or epidermis equivalent model system used, and not the source of keratinocytes. The only *in vitro* psoriasis model showing acanthosis due to increased proliferation is a commercially available model^27^ where normal human keratinocytes are seeded on a collagen gel with fibroblasts isolated from lesional psoriatic skin. This suggests that fibroblast-secreted factors, and not T-cell derived cytokines, might be a source of keratinocyte mitogens.

Histological examination of AD skin shows dermal infiltrates that are mainly comprised of Th2-helper cells that produce IL-4 and IL-13^76^. Like in psoriasis, IL-17 also plays a role in AD pathophysiology^77^. However, novel systemic therapies are aimed at the Th2-mediated inflammation and phase 3 clinical trials on a human monoclonal antibody (Dupilimab) against IL-4 receptor alpha that inhibits signaling of IL-4 and IL-13 have been completed recently^78^. IL-4 signaling reduces expression of important skin barrier proteins, like FLG, LOR, and IVL^46,79–83^, which is thought to be correlated to the impaired skin barrier function in AD^84–86^. This, however, is of recent controversy since *FLG* null mutations and a complete absence of LOR and IVL does not diminish barrier properties of HEEs^32^. We were able to generate an AD-like phenotype in our N/TERT AD-HEE by stimulating with IL-4 and IL-13 during the final three days of the air-liquid interphase culture (Fig. 5). The similar effects on N/TERT keratinocytes as compared to primary keratinocytes on downstream target genes by the herein investigated pro-inflammatory cytokines, suggest that the signalling pathways involved in the Th1, Th17, and Th2 mediated pathogenesis, like the JAK/STAT^87^ and NF-ĸB^88^ pathways are functional in both N/TERT keratinocyte cell lines.

In both N/TERT HEE disease models, established drugs targeting the epidermal keratinocyte compartment successfully reduced inflammation hallmarks suggesting a place for N/TERT keratinocytes in drug development or screening pipelines. ATRA is a widely used anti-psoriatic drug and binds to nuclear receptors, retinoic acid receptor (RAR) and retinoid X receptor (RXR) in the epidermis^89^. Coal tar activates the AHR leading to a dampened Th2 cytokine-mediated inflammatory response and the reduction of spongiosis and apoptosis^46^. Research in the field of AHR signalling has been rapidly emerging in the past years. This ligand-activated transcription factor is a master regulator of pivotal cell biological processes like xenobiotic metabolism, cell proliferation, differentiation, and migration in a variety of cells and tissues (reviewed by Esser *et al*.^90^). In the skin, the AHR regulates epidermal proliferation, differentiation, melanogenesis, wound healing, response to UV radiation, and immune responses^91^. AHR activation in N/TERT keratinocytes results in upregulated expression of known target genes *CYP1A1*, *CYP1B1*, *TIPARP* (Fig. 5A, Supplemental Fig. 6A) and negative feedback repressor gene *AHRR*, which opens up avenues to use the N/TERT cells as a convenient and unlimited cell source of human keratinocytes to study AHR-related mechanisms in the skin.

One other important field for which the N/TERT keratinocytes could be an immensely valuable asset is genome editing. The CRISPR/Cas9 system, first introduced for mammalian cell genome editing in 2013^92^, now facilitates virtually unlimited genetic manipulation and is described in studies on cell biology, disease pathophysiology, drug screening, and therapeutic interventions. *In vitro* technologies based on CRISPR/Cas9 require serial passaging of transfected cells and clonal cultures for the selection of your desired genotype. Primary cells with a limited life span are therefore not suitable for genome editing strategies and studies with CRISPR/Cas9 in skin biology or diseases remain scarce^93,94^. The unlimited lifespan, diploid nature and high similarity to primary keratinocytes make N/TERT keratinocytes the ideal cell source for genome editing studies.

Altogether, this work directs towards an expanded utilization of the N/TERT cell lines as a substitute for primary cells in biologically relevant applications in the fields of cell biology, tissue engineering, dermatology, and toxicology.

## Methods

### N/TERT keratinocytes karyotyping and short tandem repeat (STR) analysis

The N/TERT keratinocytes were karyotyped to assess chromosome abnormalities. In short, colcemid (Invitrogen, New York, USA) treated, hypotonic N/TERT keratinocytes were fixed by methanol:acetic acid (3:1) before Giemsa-staining on glass slides. Metaphases were analysed under light microscope by Clinical Assistant (RVC, Baarn, The Netherlands). Additional short tandem repeat (STR) analysis, to assess the cell lines purity, was performed by QF-PCR using the Aneufast QF-PCR kit (molGENTIX, Barcelona, Spain) according to the manufacturers protocol. Electrophoretograms were generated by use of GeneMarker (SoftGenetics LLC, Stage College, USA).

### Isolation of primary human keratinocytes

Human abdominal or breast skin was obtained from plastic surgery procedures after informed consent and in line with the principles and guidelines of the Declaration of Helsinki. Skin biopsies were taken and human primary keratinocytes were isolated as previously described^31^ and stored in liquid nitrogen until further use.

### Monolayer cultures of human primary keratinocytes and human N/TERT keratinocytes

Human primary keratinocytes (P1) were cultured in a 24 wells plate in keratinocyte growth medium (Lonza, Walkersville, USA) until near 100% cell confluence before stimulation with either Th1 (TNFα (30 ng/mL), IFNγ (500 U/mL) and IL-1α (30 ng/mL)), Th2 (IL-4 (50 ng/mL) and IL-13 (50 ng/mL)), or Th17 (IL-17 (50 ng/mL) and IL-22 (50 ng/mL)) cytokines. All cytokines were obtained from Preprotech, London, UK. Two human N/TERT keratinocyte cell lines (N/TERT-1 and N/TERT-2G), purchased from J. Rheinwald laboratory (Harvard Medical School, Boston, USA), were cultured in a 24 well plate in keratinocyte-serum free medium (K-SFM, Gibco) until 50% confluence when switched to experiment medium (50% K-SFM and 50% DF-K (DMEM:Ham’s F12, 1:1 (vol/vol) with added bovine pituitary extract (BPE, 25 µg/mL, Invitrogen,) L-glutamine (2mM, Invitrogen), EGF (0.2 ng/mL, Invitrogen) and CaCl_2_ (300 µM, Sigma-Aldrich, Saint Louis, USA), as described by Dickson *et al*.^11^. Stimulation with Th1, Th2 and Th17 cytokines (in concentrations mentioned above) was performed near 100% cell confluence. Cells were harvested 24, 48, and 72 hours post stimulation for quantitative gene expression analysis.

### Human epidermal equivalent (HEE) generation

N/TERT-HEEs were generated as previously described^32,95^ with few modifications (Supplemental Fig. 3). Briefly, inert transwells (ThinCerts, Greiner Bio-One GmbH) were coated with rat tail collagen (100 µg/mL, BD Biosciences, Bedford, USA) at 4 °C for 1 hour. 10^5^ N/TERT1 or N/TERT2G keratinocytes were seeded on the transwells in 100 µL K-SFM medium (Gibco) in a 24 wells format. After 48 hours, cultures were switched to a mixture of CnT-PR-3D medium (CELLnTEC, Bern, Switzerland) and DMEM medium (60:40 (v/v)) for 24 hours and then cultured at the air-liquid interface for 10 days. Culture medium was refreshed every other day. Optimisation of the culture protocol is shown in Supplemental Fig. 4A–C.

### Psoriasis (PS) and atopic dermatitis (AD) HEE development

N/TERT-HEEs were generated as described above (Supplemental Fig. 3A). From day 7 to 10 of the air-liquid interphase culture, PS-associated or AD-associated cytokines were added to the cultures (Supplemental Fig. 3B). For the PS model, Th1 cytokines, TNFα (5 ng/mL), IL-6 (5 ng/mL) and IL-1α (10 ng/mL) or Th17 cytokines IL-17 (30 ng/mL), and IL-22 (30 ng/mL) were used. The AD model was generated by adding the Th2 cytokine IL-4 (10 ng/mL) to the culture media. The PS or AD phenotype in HEEs was rescued by addition of all trans retinoic acid (ATRA, 1 µM, Sigma-Aldrich) or a coal tar extract (prepared from crude pix lithanthracis, Fagron BV, Capelle aan den IJssel, Netherlands), respectively at day 8 of the culture experiment (Supplemental Fig. 3C). At day 10, N/TERT-HEEs were harvested for gene expression analysis and immunohistochemistry.

### Total RNA isolation and quantitative real-time polymerase chain reaction

Total RNA was isolated using the Favorprep total tissue RNA kit (Favorgen Biotech, Taiwan), according to the manufacturers protocol. cDNA was generated, after DNase treatment, and used for quantitative real-time PCR (qPCR) by use of the MyiQ Single-Colour Real-Time Detection System (Bio-Rad laboratories, Hercules, USA) for quantification with Sybr Green and melting curve analysis. All primers (Table 2) were obtained from Biolegio (Nijmegen, The Netherlands). Target gene expression levels were normalized to the expression of human *acidic ribosomal phosphoprotein P0* (*RPLP0*). The relative expression levels of all genes of interest were measured using the 2^−ΔΔCT^ method^96^.

**Table 2:** Primer sequences of all primers.

Gene	Name	Forward primer (5′–3′)	Reverse primer (5′–3′)	***E*** ^*^
*FLG*	Filaggrin	acttcactgagtttcttctgatggtatt	tccagacttgagggtctttttctg	1.89
*LOR*	Loricrin	aggttaagacatgaaggatttgcaa	ggcaccgatgggcttagag	2.08
*IVL*	Involucrin	acttatttcgggtccgctaggt	gagacatgtagagggacagagtcaag	1.93
*HRNR*	Hornerin	tacaaggcgtcatcactgtcatc	atctggatcgtttggattcttcag	2.12
*LCE1A*	Late cornified envelope 1 A	tgcaagagtggctgagatgc	agacaacacagttggtgtcagg	2.18
*KRT1*	Keratin 1	gatgaaatcaacaagcggacaa	tggtagagtgctgtaaggaaatcaatt	2.24
*KRT5*	Keratin 5	atctctgagatgaaccggatgatc	cagattggcgcactgtttctt	2.26
*KRT14*	Keratin 14	ggcctgctgagatcaaagactac	cactgtggctgtgagaatcttgtt	1.93
*KRT16*	Keratin 16	gatcattgcggccaccat	tgctcatacttggtcctgaagtca	2.01
*TGM1*	Transglutaminase 1	cccccgcaatgagatctaca	atcctcatggtccacgtacaca	1.99
*TGM3*	Transglutaminase 3	ggaaggactctgccacaatgtc	tgtctgacttcaggtacttctcatactg	2.06
*CST6*	Cystatin M/E	tccgagacacgcacatcatc	ccatctccatcgtcaggaagtac	1.98
*CA2*	Carbonic anhydrase II	aacaatggtcatgctttcaacg	tgtccatcaagtgaaccccag	2.02
*CCL26*	C-C motif chemokine ligand 26	tcattcagtaaagaggcgaagtattatc	cagttttttggagggcatctg	1.89
*DEFB4*	Human beta defensin 2	gatgcctcttccaggtgttttt	ggatgacatatggctccactctt	1.99
*PI3*	Skin-derived antileukoprotease	catgagggccagcagctt	tttaacaggaactcccgtgaca	2.02
*CYP1A1*	Cytochrome p450 family 1A1	ctggagaccttccgacactctt	gtaaaagcctttcaaacttgtgtctct	2.02
*CYP1B1*	Cytochrome p450 family 1B1	tggctgctcctcctcttcac	ccacgacctgatccaattctg	2.02
*TIPARP*	TCDD inducible polymerase	aagctcctccacctcttgaa	tctgcagaaacagggacttg	2.05
*AHRR*	Aryl hydrocarbon receptor repressor	aaggctgctgttggagtctcttaat	gatgtagtcataaatgttctggtgcat	2.15
*RPLP0*	Ribosomal protein P0	caccattgaaatcctgagtgatgt	tgaccagcccaaaggagaag	2.02

*E is efficiency as fold increase in fluorescence per PCR cycle.

### Morphological and immunohistochemical analysis

HEEs were fixed in 4% formalin solution for 4 hours and subsequently embedded in paraffin. 6 µm sections were stained with hematoxylin and eosin (H&E, Sigma-Aldrich) or processed for immunohistochemical analysis. Sections were blocked for 15 minutes with 5% normal goat, rabbit, or horse serum in phosphate-buffered saline (PBS) and subsequently incubated with the antibodies mouse anti-FLG, rabbit anti-LOR, mouse anti-IVL, mouse anti-Ki67, goat anti-hBD2, or rabbit anti-SKALP/Elafin for 1 hour at room temperature. Next, a 30 minute incubation step with biotinylated rabbit anti-goat, horse anti-mouse, or goat anti-rabbit (Vector Laboratories, Burlingame, USA) was performed, followed by a 30 minute incubation with avidin-biotin complex (Vector laboratories). The peroxidase activity of 3-Amino-9-ethylcarbazole (AEC) was used to visualize the protein expression and the sections were mounted using glycerol gelatin (Sigma-Aldrich). See Table 3 for details on the antibodies used.

**Table 3:** Antibodies used for immunohistochemistry.

Antibody; clone	Manufacturer	Dilution
FLG; clone 15C10	Leica Biosystems, Newcastle, UK	1:100
LOR; Covance 145P-100	Covance Inc., Princeton, USA	1:4000
IVL; Mon150	Van Duijnhoven *et al*.^97^	1:20
Ki67; MIB-1	DAKO, Heverlee, Belgium	1:50
pAb to beta 2 defensin	Abcam, Cambridge, UK	1:100
SKALP/Elafin; 92-1	Schalkwijk *et al*.^33^	1:500

### Lucifer yellow dye penetration assay

To study the outside-in *stratum corneum* barrier function, Lucifer Yellow (1 mM, Sigma-Aldrich) was applied on top of the HEEs and was allowed to incubate for 60 minutes, in the dark, at room temperature^32,58^. HEEs were fixated in buffered 4% formalin solution, embedded in paraffin and sectioned. 6 µm sections were deparaffinised and mounted with Fluoromount-G, containing DAPI (eBioscience Inc. San Diego, USA). SDS treatment of the HEEs as a positive control for the Lucifer Yellow penetration upon skin barrier disruption was shown in a previous study by our group^32^.

### Biotin penetration assay

To study the inside-out *stratum corneum* barrier function, the HEEs were turned upside down and EZ-link sulfo-NHS-LC-biotin (3.3 mg/mL, Thermo Fisher Scientific, Waltham, USA) was applied on the bottom of the filters and allowed to incubate for 60 minutes at room temperature^32^. HEEs were fixated in buffered 4% formalin solution, embedded in paraffin and sectioned. 6 µm sections were deparaffinised and incubated for 30 minutes, in the dark, with 1:200 Alexa Fluor 594 streptavidin (Thermo Fisher Scientific) conjugate. The sections were mounted with Fluoromount-G containing DAPI.

### Statistics

Data are represented as mean ± SEM of at least three technical (N/TERT keratinocytes) or biological (primary keratinocytes) replicates. Raw ΔCt values were used to statistically analyze the quantitative PCR results using the commercially available SPSS software (IBM SPSS Statistics 22). One-way analysis of variance followed by Bonferroni post hoc testing was performed. *P* < 0.05 was considered to be of statistical significance.

### Literature search

A search of relevant literature that reports the use of N/TERT1 and/or N/TERT2G cell lines has been conducted in April 2017. A first extensive literature search was performed by investigating all publications that cited the original N/TERT paper by Dickson *et al*.^11^. From these 229 publications, the abstract and materials and methods section was evaluated for the use of N/TERT1 and N/TERT2G cell lines. This led to a total of 58 papers, of which 42 studies used the cells in conventional monolayer culture systems (Supplemental Table 1) and 16 studies used the cells to generate skin- or epidermal equivalents (Table 1). Furthermore, we used Pubmed and Google Scholar and the search terms included ‘N/TERT’, ‘N/TERT1’, ‘N/TERT2G’, ‘TERT’, ‘TRT’, ‘keratinocytes’, ‘epidermal equivalent’, and ‘skin equivalent’.

### Data availability

The datasets generated during and/or analysed during the current study are available from the corresponding author on request.

## Electronic supplementary material

Supplementary information
