# Supplementary material for: Immortalized N/TERT keratinocytes as an alternative cell source in 3D human epidermal models
Source: Sci Rep. 2017 Sep 19;7:11838. doi: 10.1038/s41598-017-12041-y (PMC5605545; doi:10.1038/s41598-017-12041-y)
Supplement: Supplementary file 1 — Supplementary information [file 41598_2017_12041_MOESM1_ESM.pdf]

SUPPLEMENTAL MATERIAL

Immortalized N/TERT keratinocytes as an alternative cell source in 3D human epidermal models

Jos P.H. Smits[1], Hanna Niehues[1], Gijs Rikken[1], Ivonne M.J.J. van Vlijmen-Willems[1], Guillaume W.H.J.F.L. van de Zande[2], Patrick L.J.M. Zeeuwen[1], Joost Schalkwijk[1], Ellen H. van den Bogaard[1]

[1]Department of Dermatology, Radboud Institute for Molecular Life Sciences (RIMLS), Radboud University Medical Center (Radboudumc), PO BOX 9101, 6500 HB Nijmegen, The Netherlands.

[2]Department of Human Genetics, Radboud University Medical Center (Radboudumc), PO BOX 9101, 6500 HB Nijmegen, The Netherlands.

Corresponding author: E.H. van den Bogaard, Department of Dermatology, Radboud Institute for Molecular Life Sciences (RIMLS), Radboud University Medical Center (Radboudumc), PO BOX 9101, 6500 HB Nijmegen, The Netherlands. E: ellen.vandenbogaard@radboudumc.nl, T: 0031-243614093

Supplemental Figures

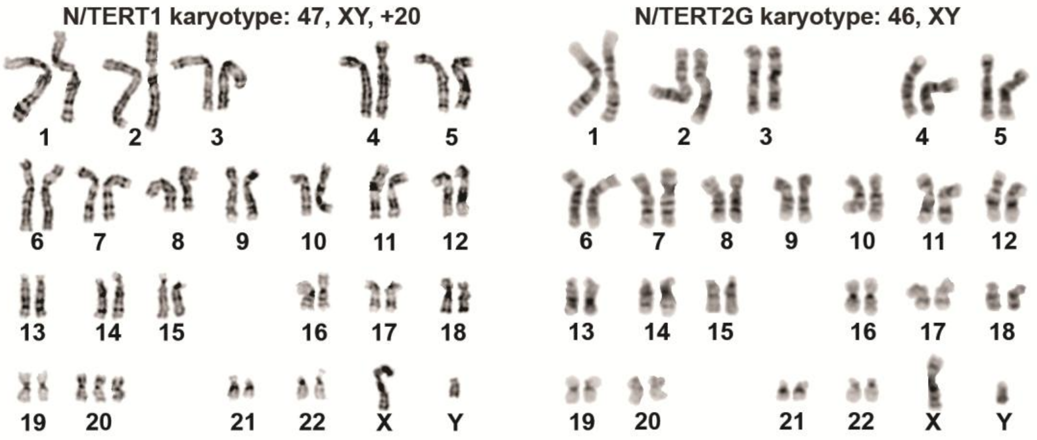

Supplemental figure 1. Karyogram of N/TERT keratinocytes. Karyogram of N/TERT keratinocytes showing G-bands after Giemsa-staining.

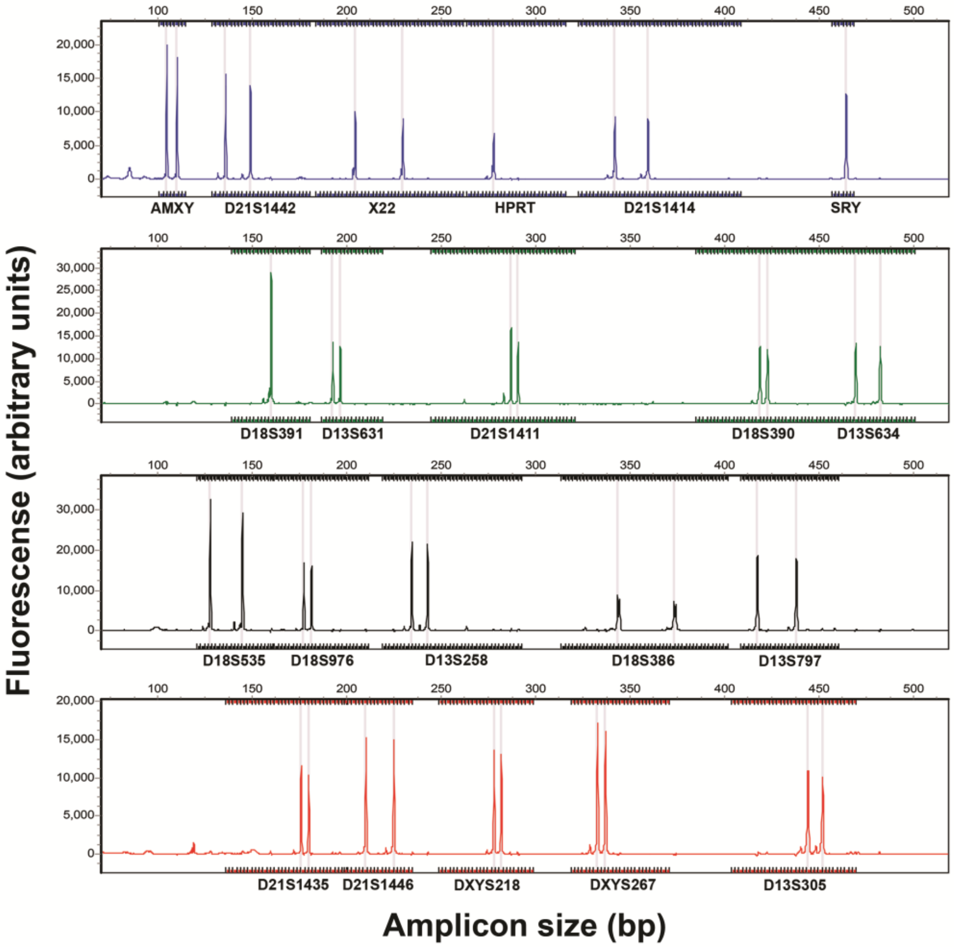

Supplemental figure 2. Short tandem repeat analysis of N/TERT1 keratinocytes. QF-PCR based short tandem repeat analysis of N/TERT1 keratinocytes.

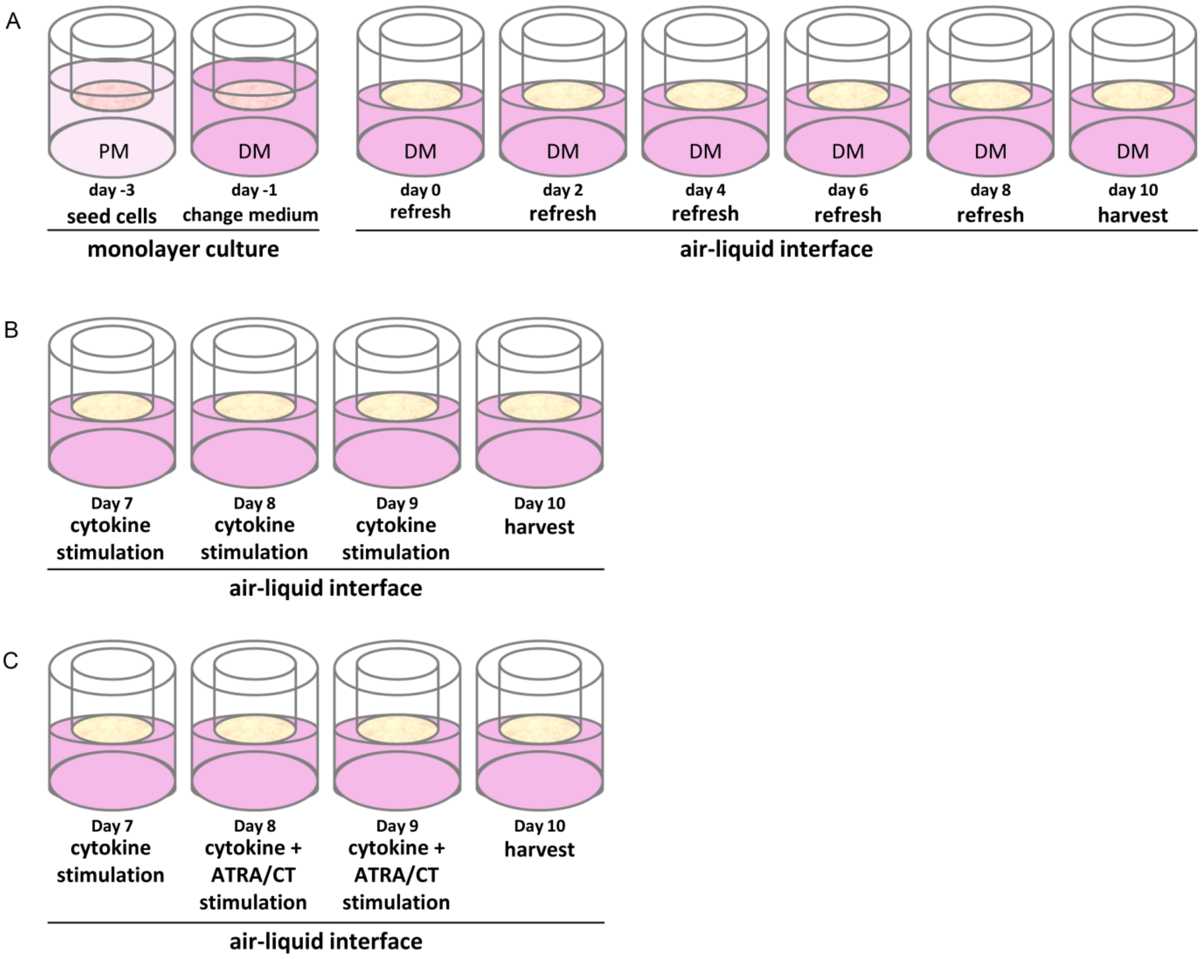

Supplemental figure 3. Schematic overview of N/TERT keratinocyte-based HEE model culture. A) Culture protocol for normal N/TERT1 and N/TERT2G HEEs. B) Culture protocol for inflammatory N/TERT1 HEEs from day 7 onwards. C) Culture protocol for inflammatory N/TERT1 HEEs and all trans retinoic acid (ATRA) or coal tar (CT) treatment from day 7 onwards.

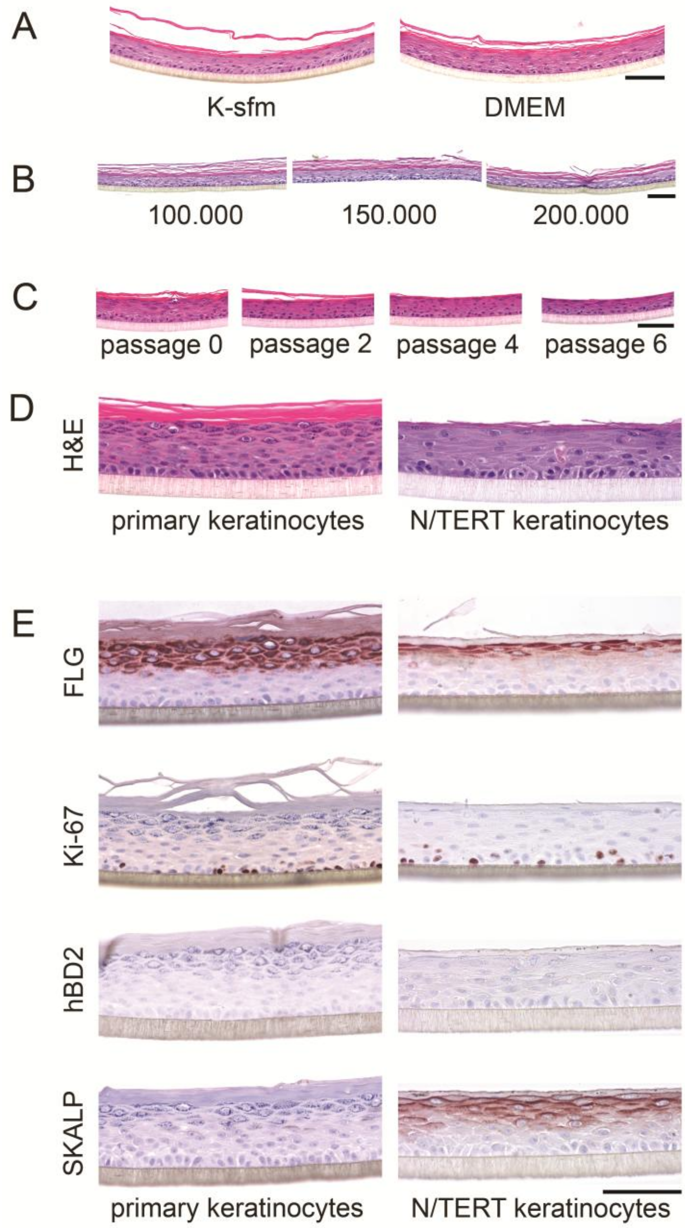

Supplemental figure 4. Optimisation of the N/TERT1 keratinocyte-based HEE model. HEEs cultured A) with either K-SFM or DMEM medium mixed with CnT-PR-3D medium (60:40 ratio), B) with varying seeding densities, and C) after the N/TERT1 cells were passaged up to 6 times were visualized by haematoxylin eosin (HE). D) Primary keratinocyte based HEEs and N/TERT1 keratinocyte-based HEEs are highly similar based on morphology and E) terminal differentiation, proliferation, and host defense proteins expression, as visualized by immunohistochemistry. Scale bar=100 µm.

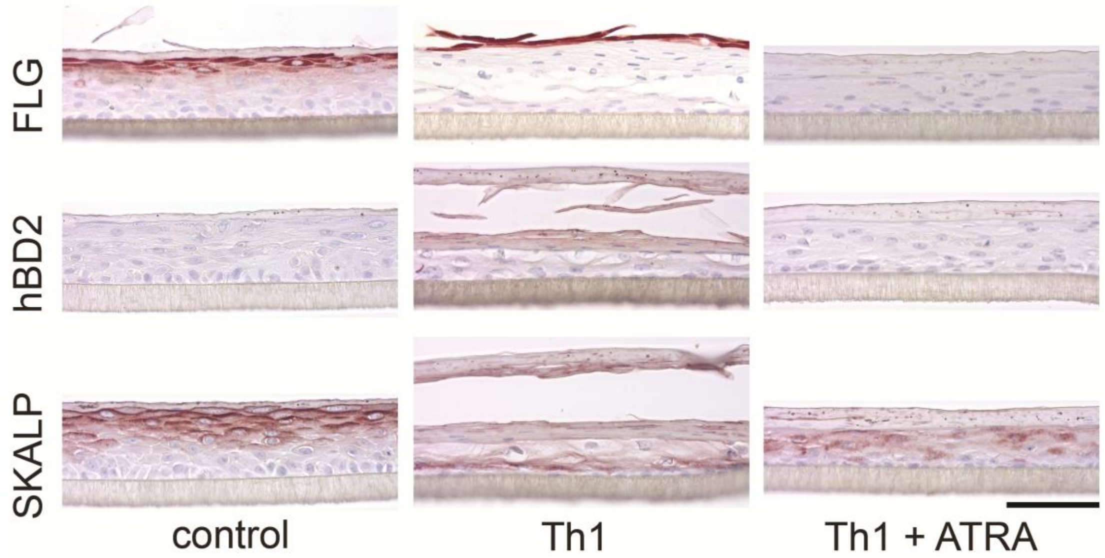

Supplemental figure 5. N/TERT1 keratinocytes can be used to generate a psoriasis-like HEE (PS-HEE) disease model. N/TERT1 HEEs were harvested for morphological analysis after stimulation with Th1 cytokines (TNFα, IL-6, and IL-1α) and rescued by addition of all trans retinoic acid (ATRA). Scale bar=100 µm.

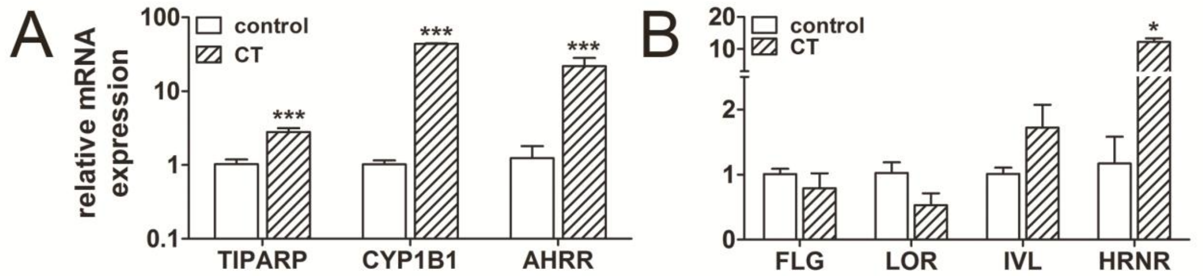

Supplemental figure 6. N/TERT1 keratinocytes possess the ability to respond in an AHR-mediated manner. N/TERT1 HEEs were harvested for gene expression analysis after stimulation with coal tar Bars represent mean±SEM. *p<0.05 ***p<0.001 relative to control unstimulated keratinocytes.

Supplemental table 1: Studies using N/TERT keratinocytes in conventional monolayer culture experiments.

**Author**	**Field of study / keywords**
Dickson *et al.*[1$]	Initial paper describing N/TERT keratinocytes
Ozbun[2]	Human papillomavirus
Natarajan *et al.*[3]	Skin cancer, squamous cell carcinoma
Patterson *et al.*[4]	Human papillomavirus
Hardman *et al.* [5]	Epidermal differentiation
Natarajan *et al.*[6]	Wound healing
Burdick *et al.*[7]	Cellular signalling
Lewis *et al.*[8]	UV radiation
Eichberger *et al.*[9]	Cellular signalling
Utikal *et al.*[10]	Induced pluripotent stemcells
Warters *et al.*[11]	Ionizing radiation
Konger *et al.* [12]	UV radiation
Teh *et al.*[13]	UV radiation, skin cancer
Wei *et al.* [14]	Cellular signaling
Yi *et al.* [15]	Cellular signaling
Oliver *et al.*[16]	Human papillomavirus
Di *et al.*[17]	Gene therapy
Jackson *et al.*[18]	Epidermal differentiation
Weir *et al.*[19]	Cellular signaling
Chikh *et al.*[20]	Epidermal differentiation
Bose *et al.*[21]	Epidermal differentiation
Di *et al.*[22]	Gene therapy, epidermal differentiation
White *et al.*[23]	Human papillomavirus
Degen *et al.*[24]	Skin cancer, squamous cell carcinoma
Stoll *et al.*[25]	Wound healing
Cho *et al*[26]	Skin cancer
White *et al.*[27]	Human papillomavirus
Wei *et al.*[28]	Tissue generation
Liu *et al.*[29]	UV radiation
Bause *et al.*[30]	Epidermal differentiation
Nguyen *et al.*[31]	Skin cancer, cell signaling
Abaitua *et al.*[32]	Cell migration, herpes simplex virus
Dreesen *et al.*[33]	Epidermal proliferation
Dreesen *et al.*[34]	Cellular aging and disease
Johns *et al.*[35]	Cell signaling, herpes simplex virus
Pantazi *et al.*[36]	Apoptosis
Chikh *et al.* [37]	Apoptosis
Valdimara *et al.*[38]	Human papillomavirus
Bigliardi *et al.*[39]	Wound healing
Loesch *et al.*[40]	UV radiation
Kaseb *et al.*[41]	Cancer, head and neck squamous cell carcinoma
Sayers *et al.*[42]	Herpes simplex virus
Chen *et al.*[43]	Cellular signaling

[$]Initial paper describing the N/TERT cell line development; UV: ultraviolet.

Supplemental references

1 Dickson, M. A., Hahn, W. C., Ino, Y. *et al.* Human keratinocytes that express hTERT and also bypass a p16(INK4a)-enforced mechanism that limits life span become immortal yet retain normal growth and differentiation characteristics. *Molecular and cellular biology* **20**, 1436-1447 (2000).

2 Ozbun, M. A. Human papillomavirus type 31b infection of human keratinocytes and the onset of early transcription. *Journal of virology* **76**, 11291-11300 (2002).

3 Natarajan, E., Saeb, M., Crum, C. P. *et al.* Co-expression of p16(INK4A) and laminin 5 gamma2 by microinvasive and superficial squamous cell carcinomas in vivo and by migrating wound and senescent keratinocytes in culture. *The American journal of pathology* **163**, 477-491 (2003).

4 Patterson, N. A., Smith, J. L. & Ozbun, M. A. Human papillomavirus type 31b infection of human keratinocytes does not require heparan sulfate. *Journal of virology* **79**, 6838-6847, doi:10.1128/JVI.79.11.6838-6847.2005 (2005).

5 Hardman, M. J., Liu, K., Avilion, A. A. *et al.* Desmosomal cadherin misexpression alters beta-catenin stability and epidermal differentiation. *Molecular and cellular biology* **25**, 969-978, doi:10.1128/MCB.25.3.969-978.2005 (2005).

6 Natarajan, E., Omobono, J. D., 2nd, Guo, Z. *et al.* A keratinocyte hypermotility/growth-arrest response involving laminin 5 and p16INK4A activated in wound healing and senescence. *The American journal of pathology* **168**, 1821-1837, doi:10.2353/ajpath.2006.051027 (2006).

7 Burdick, A. D., Bility, M. T., Girroir, E. E. *et al.* Ligand activation of peroxisome proliferator-activated receptor-beta/delta(PPARbeta/delta) inhibits cell growth of human N/TERT-1 keratinocytes. *Cellular signalling* **19**, 1163-1171, doi:10.1016/j.cellsig.2006.12.007 (2007).

8 Lewis, D. A., Yi, Q., Travers, J. B. *et al.* UVB-induced senescence in human keratinocytes requires a functional insulin-like growth factor-1 receptor and p53. *Molecular biology of the cell* **19**, 1346-1353, doi:10.1091/mbc.E07-10-1041 (2008).

9 Eichberger, T., Kaser, A., Pixner, C. *et al.* GLI2-specific transcriptional activation of the bone morphogenetic protein/activin antagonist follistatin in human epidermal cells. *The Journal of biological chemistry* **283**, 12426-12437, doi:10.1074/jbc.M707117200 (2008).

10 Utikal, J., Polo, J. M., Stadtfeld, M. *et al.* Immortalization eliminates a roadblock during cellular reprogramming into iPS cells. *Nature* **460**, 1145-1148, doi:10.1038/nature08285 (2009).

11 Warters, R. L., Packard, A. T., Kramer, G. F. *et al.* Differential gene expression in primary human skin keratinocytes and fibroblasts in response to ionizing radiation. *Radiation research* **172**, 82-95, doi:10.1667/RR1677.1 (2009).

12 Konger, R. L., Martel, K. C., Jernigan, D. *et al.* The peroxisome proliferator-activated receptor gamma system regulates ultraviolet B-induced prostaglandin e(2) production in human epidermal keratinocytes. *PPAR research* **2010**, 467053, doi:10.1155/2010/467053 (2010).

13 Teh, M. T., Gemenetzidis, E., Chaplin, T. *et al.* Upregulation of FOXM1 induces genomic instability in human epidermal keratinocytes. *Molecular cancer* **9**, 45, doi:10.1186/1476-4598-9-45 (2010).

14 Wei, Q., Hariharan, V. & Huang, H. Cell-cell contact preserves cell viability via plakoglobin. *PloS one* **6**, e27064, doi:10.1371/journal.pone.0027064 (2011).

15 Yi, Q., Zhao, X., Huang, Y. *et al.* p53 dependent centrosome clustering prevents multipolar mitosis in tetraploid cells. *PloS one* **6**, e27304, doi:10.1371/journal.pone.0027304 (2011).

16 Oliver, A. W., He, X., Borthwick, K. *et al.* The HPV16 E6 binding protein Tip-1 interacts with ARHGEF16, which activates Cdc42. *British journal of cancer* **104**, 324-331, doi:10.1038/sj.bjc.6606026 (2011).

17 Di, W. L., Larcher, F., Semenova, E. *et al.* Ex-vivo gene therapy restores LEKTI activity and corrects the architecture of Netherton syndrome-derived skin grafts. *Molecular therapy : the*

journal of the American Society of Gene Therapy **19**, 408-416, doi:10.1038/mt.2010.201 (2011).
18 Jackson, B., Brown, S. J., Avilion, A. A. *et al.* TALE homeodomain proteins regulate site-specific terminal differentiation, LCE genes and epidermal barrier. *Journal of cell science* **124**, 1681-1690, doi:10.1242/jcs.077552 (2011).
19 Weir, L., Robertson, D., Leigh, I. M. *et al.* Hypoxia-mediated control of HIF/ARNT machinery in epidermal keratinocytes. *Biochimica et biophysica acta* **1813**, 60-72, doi:10.1016/j.bbamcr.2010.11.014 (2011).
20 Chikh, A., Matin, R. N., Senatore, V. *et al.* iASPP/p63 autoregulatory feedback loop is required for the homeostasis of stratified epithelia. *The EMBO journal* **30**, 4261-4273, doi:10.1038/emboj.2011.302 (2011).
21 Bose, A., Teh, M. T., Hutchison, I. L. *et al.* Two mechanisms regulate keratin K15 expression in keratinocytes: role of PKC/AP-1 and FOXM1 mediated signalling. *PloS one* **7**, e38599, doi:10.1371/journal.pone.0038599 (2012).
22 Di, W. L., Semenova, E., Larcher, F. *et al.* Human involucrin promoter mediates repression-resistant and compartment-specific LEKTI expression. *Human gene therapy* **23**, 83-90, doi:10.1089/hum.2011.091 (2012).
23 White, E. A., Sowa, M. E., Tan, M. J. *et al.* Systematic identification of interactions between host cell proteins and E7 oncoproteins from diverse human papillomaviruses. *Proceedings of the National Academy of Sciences of the United States of America* **109**, E260-267, doi:10.1073/pnas.1116776109 (2012).
24 Degen, M., Natarajan, E., Barron, P. *et al.* MAPK/ERK-dependent translation factor hyperactivation and dysregulated laminin gamma2 expression in oral dysplasia and squamous cell carcinoma. *The American journal of pathology* **180**, 2462-2478, doi:10.1016/j.ajpath.2012.02.028 (2012).
25 Stoll, S. W., Rittie, L., Johnson, J. L. *et al.* Heparin-binding EGF-like growth factor promotes epithelial-mesenchymal transition in human keratinocytes. *The Journal of investigative dermatology* **132**, 2148-2157, doi:10.1038/jid.2012.78 (2012).
26 Cho, Y. Y., Lee, M. H., Lee, C. J. *et al.* RSK2 as a key regulator in human skin cancer. *Carcinogenesis* **33**, 2529-2537, doi:10.1093/carcin/bgs271 (2012).
27 White, E. A. & Howley, P. M. Proteomic approaches to the study of papillomavirus-host interactions. *Virology* **435**, 57-69, doi:10.1016/j.virol.2012.09.046 (2013).
28 Wei, Q., Reidler, D., Shen, M. Y. *et al.* Keratinocyte cytoskeletal roles in cell sheet engineering. *BMC biotechnology* **13**, 17, doi:10.1186/1472-6750-13-17 (2013).
29 Liu, K., Yu, D., Cho, Y. Y. *et al.* Sunlight UV-induced skin cancer relies upon activation of the p38alpha signaling pathway. *Cancer research* **73**, 2181-2188, doi:10.1158/0008-5472.CAN-12-3408 (2013).
30 Bause, A. S., Matsui, M. S. & Haigis, M. C. The protein deacetylase SIRT3 prevents oxidative stress-induced keratinocyte differentiation. *The Journal of biological chemistry* **288**, 36484-36491, doi:10.1074/jbc.M113.472324 (2013).
31 Nguyen, H. B., Babcock, J. T., Wells, C. D. *et al.* LKB1 tumor suppressor regulates AMP kinase/mTOR-independent cell growth and proliferation via the phosphorylation of Yap. *Oncogene* **32**, 4100-4109, doi:10.1038/onc.2012.431 (2013).
32 Abaitua, F., Zia, F. R., Hollinshead, M. *et al.* Polarized cell migration during cell-to-cell transmission of herpes simplex virus in human skin keratinocytes. *Journal of virology* **87**, 7921-7932, doi:10.1128/JVI.01172-13 (2013).
33 Dreesen, O., Chojnowski, A., Ong, P. F. *et al.* Lamin B1 fluctuations have differential effects on cellular proliferation and senescence. *The Journal of cell biology* **200**, 605-617, doi:10.1083/jcb.201206121 (2013).
34 Dreesen, O., Ong, P. F., Chojnowski, A. *et al.* The contrasting roles of lamin B1 in cellular aging and human disease. *Nucleus* **4**, 283-290, doi:10.4161/nucl.25808 (2013).

35 Johns, H. L., Gonzalez-Lopez, C., Sayers, C. L. *et al.* Rab6 dependent post-Golgi trafficking of HSV1 envelope proteins to sites of virus envelopment. *Traffic* **15**, 157-178, doi:10.1111/tra.12134 (2014).

36 Pantazi, E., Gemenetzidis, E., Trigiante, G. *et al.* GLI2 induces genomic instability in human keratinocytes by inhibiting apoptosis. *Cell death & disease* **5**, e1028, doi:10.1038/cddis.2013.535 (2014).

37 Chikh, A., Sanza, P., Raimondi, C. *et al.* iASPP is a novel autophagy inhibitor in keratinocytes. *Journal of cell science* **127**, 3079-3093, doi:10.1242/jcs.144816 (2014).

38 Vieira, V. C., Leonard, B., White, E. A. *et al.* Human papillomavirus E6 triggers upregulation of the antiviral and cancer genomic DNA deaminase APOBEC3B. *mBio* **5**, doi:10.1128/mBio.02234-14 (2014).

39 Bigliardi, P. L., Neumann, C., Teo, Y. L. *et al.* Activation of the delta-opioid receptor promotes cutaneous wound healing by affecting keratinocyte intercellular adhesion and migration. *British journal of pharmacology* **172**, 501-514, doi:10.1111/bph.12687 (2015).

40 Loesch, M. M., Collier, A. E., Southern, D. H. *et al.* Insulin-like growth factor-1 receptor regulates repair of ultraviolet B-induced DNA damage in human keratinocytes in vivo. *Molecular oncology* **10**, 1245-1254, doi:10.1016/j.molonc.2016.06.002 (2016).

41 Kaseb, H. O., Fohrer-Ting, H., Lewis, D. W. *et al.* Identification, expansion and characterization of cancer cells with stem cell properties from head and neck squamous cell carcinomas. *Experimental cell research* **348**, 75-86, doi:10.1016/j.yexcr.2016.09.003 (2016).

42 Sayers, C. L. & Elliott, G. Herpes Simplex Virus 1 Enters Human Keratinocytes by a Nectin-1-Dependent, Rapid Plasma Membrane Fusion Pathway That Functions at Low Temperature. *Journal of virology* **90**, 10379-10389, doi:10.1128/JVI.01582-16 (2016).

43 Chen, W., Kinsler, V. A., Macmillan, D. *et al.* Tissue Kallikrein Inhibitors Based on the Sunflower Trypsin Inhibitor Scaffold - A Potential Therapeutic Intervention for Skin Diseases. *PloS one* **11**, e0166268, doi:10.1371/journal.pone.0166268 (2016).
